# Regulated cell death pathways in doxorubicin-induced cardiotoxicity

**DOI:** 10.1038/s41419-021-03614-x

**Published:** 2021-04-01

**Authors:** Effimia Christidi, Liam R. Brunham

**Affiliations:** 1 Centre for Heart Lung Innovation, Department of Medicine, University of British Columbia, Vancouver, BC Canada; 2 Department of Medicine, University of British Columbia, Vancouver, BC Canada; 3 Department of Medical Genetics, University of British Columbia, Vancouver, BC Canada

**Keywords:** Mechanisms of disease, Cardiomyopathies

## Abstract

Doxorubicin is a chemotherapeutic drug used for the treatment of various malignancies; however, patients can experience cardiotoxic effects and this has limited the use of this potent drug. The mechanisms by which doxorubicin kills cardiomyocytes has been elusive and despite extensive research the exact mechanisms remain unknown. This review focuses on recent advances in our understanding of doxorubicin induced regulated cardiomyocyte death pathways including autophagy, ferroptosis, necroptosis, pyroptosis and apoptosis. Understanding the mechanisms by which doxorubicin leads to cardiomyocyte death may help identify novel therapeutic agents and lead to more targeted approaches to cardiotoxicity testing.

## Facts

- Doxorubicin simultaneously triggers different regulated cell death (RCD) pathways.
- The role of apoptosis in doxorubicin induced cardiotoxicity (DIC) is well established, however new molecular players are constantly emerging.
- In recent years, emerging RCD pathways have been implicated in DIC including ferroptosis, necroptosis and pyroptosis.
- The role of autophagy remains unclear as to whether it has a protective or detrimental effect on doxorubicin response.
- Sirtuins protect from DIC and are involved in multiple RCDs.

## Open questions

- What is the effect of genetic variants associated with DIC on regulated cell death?
- Which RCD pathway has the strongest effect on doxorubicin induced cardiotoxicity?
- Are there therapeutic agents that act upstream that can simultaneously block more than one RCD pathways?

## Introduction

Advancements in drug discovery have resulted in a significant increase in survivorship for patients with cancer^1^. However, many chemotherapy drugs cause adverse drug reactions with cardiovascular toxicity being one of the most common and life threatening^2^. Anthracyclines are a class of chemotherapeutic drugs administered in adult and pediatric patients for the treatment of malignancies such as lymphoma, sarcoma, breast cancer, and others^3^. The most commonly used anthracycline is doxorubicin. However, despite its potency, up to a quarter of patients experience doxorubicin-induced cardiotoxicity (DIC), limiting the use of this agent^4^. Clinically, DIC is characterized by a reduction in the left ventricular ejection fraction, an increase in the ventricular wall thickness, arrhythmia, and heart failure which can result in death^3,5^.

Despite extensive research on the mechanisms by which doxorubicin causes cardiotoxicity, the molecular pathogenesis of DIC remains incompletely understood^3,6,7^. The three major sources of cell damage that lead to cardiomyocyte cell death are (1) excessive reactive oxygen species (ROS) production leading to damage to lipids, DNA and proteins; (2) TOP2B poisoning creating double-strand breaks; and (3) mitochondria damage^8^.

Recently, novel regulated cell death (RCD) pathways have been described^9,10^ and new research has emerged identifying the role of these pathways in DIC. Here we review recent discoveries of the role of RCD pathways in DIC in the context of autophagy, necroptosis, ferroptosis, pyroptosis, and apoptosis and we discuss how these findings have advanced our understanding of the molecular mechanisms underlying DIC.

## Regulated cell death

Initially, cell death was considered a passive result of uncontrolled cellular damage. Subsequent discoveries led to the recognition that cells can die in a genetically and biochemically coordinated manner. In 1972, apoptosis—the first form of RCD—was described^11^. There are now more than ten different types of RCD, defined as precise signaling pathways performed by distinct molecules with specific biochemical and functional consequences^9^. RCD differs from accidental, unregulated cell death (necrosis) which is triggered unexpectedly by cellular injury or attack and lacks controlled signaling mechanisms^9^. Doxorubicin can induce cardiomyocyte death via both regulated and unregulated cell death^9^.

## Autophagy

Macroautophagy (hereafter referred to as autophagy) is a homeostatic process by which cellular components are degraded and recycled under normal and stress conditions^12^. Such cellular stress conditions are caused by doxorubicin and as might be expected autophagy may be activated during doxorubicin treatment. Indeed, doxorubicin can trigger autophagy, but it is the deregulation of autophagy that leads to excessive cardiomyocyte death^13^.

Autophagy begins with activation of the AMPK pathway and the inhibition of the mTOR pathway, which signals the budding of the pre-autophagosome from the endoplasmic reticulum and proceeds with the formation of an initiation complex consisting of Unc-51-like kinase 1 (ULK1), RB1-inducible coiled-coil protein 1 (FIP200), and autophagy-related gene (Atg) 13^14^. Nucleation and recruitment of many tethering proteins then take places such as Beclin 1, which is then phosphorylated by ULK1, and which in turn activates vacuolar protein sorting 34 (Vps34) and Vps15. Vps 34-15 then recruits multiple Atg proteins and leads to the formation of the autophagosome^14^. Subsequently, the autophagosome maturates and elongates. At this stage, LC3-I (microtubule-associated protein 1A/1B-light chain 3) a key protein in autophagosome biosynthesis becomes lipidated to form mature LC3-II and protein p62, a ubiquitin-binding cargo receptor, that sorts proteins, organelles, and other aggregates within the autophagosome^14^. Finally, in the last step of autophagy, the autophagosome fuses with the lysosome, where proteases breakdown the autophagosome and its compartments, leading to the degradation or the recycling of damaged components^15^.

Whether doxorubicin induces or disrupts autophagy in cardiac tissue is controversial as studies examining the role of autophagy in DIC have reported conflicting evidence (Table 1). For example, in the initiation stage, studies report that doxorubicin upregulates AMPK^16–20^, while others showed no change^16,21,22^ or a decrease in AMPK activation^23–25^. Similarly, pharmacologic or genetic inhibition of autophagy has yielded contradictory results with some studies reporting protection while others show that blockade of autophagy results in DIC attenuation (Table 1)^19,26–30^.

**Table 1:** Evidence of doxorubicin-induced autophagy in the heart.

Model	Targets	Autophagy	Treatment/Effect on DIC	References
NRCM; C57BL/6J mice	AMPK ↑ LC3-II ↑ Atg 5, 6, 8, 12 ↑	Increase	Ghrelin/ protection; 3-MA/ protection; Compound C/ protection	^17^
H9c2; MEFs	AMPK ↓ Sirt1 dysfunction	Decrease	Genetic deletion of AMPK/susceptibility; Resveratrol/ protection	^24^
C57BL/6J mice; NRCM; H9Cc2	LC3-II ↑ lysosomal cathepsins ↓	Decrease	Beclin 1+/−/protection Beclin 1 overexpression/susceptibility BafA1/ protection	^30^
SD rats; ARCM; AMCM; NRCM; H9Cc2	TFEB ↓ Cathepsin B ↓	Decrease	TFEB overexpression/protection Torin 1/protection	^33^
NRCM	mTOR activation; Beclin 1 inhibition	Decrease	3-MA/ susceptibility; siRNA-Beclin 1/susceptibility; rapamycin/protection	^122^
H9c2 cells, C57BL/6J mice	Beclin 1 ↑ LC3II/I ↑	Increase	Ophiopogonin-D/protection; 3-MA/protection	^123^
NRCMs	GATA4 ↓ Bcl-2 ↓ LC3-II ↑ autophagy flux ↑	Increase	3-MA/protection; Rapamycin/susceptibility	^124^
ARVMs	LC3-I, LC3-II ↑ Lysosomal acidification ↓	Decrease	CQ/susceptibility	^32^
SD rats; NRCM	Beclin 1 ↑	Increased	3-MA/ protection	^125^
NRCM	Beclin 1 ↑ Atg5 ↑ Atg 12 ↑	Increase	Resveratrol/ protection; 3-MA/ protection; shBeclin 1/ protection	^126^
C57BL/6J mice	Nrf-2 ↑ LC3-I, LC3-II ↑ p62 ↑ Autophagic flux ↓	Decrease	Nrf2*-/-* / susceptibility Nrf2 overexpression/ protection BafA1/ susceptibility	^127^
SD Rat; NRCM; H9c2	AMPK ↓ mTOR ↑ LC3-II ↓ Autophagic flux ↓	Decrease	Astragalus polysaccharides/ protection	^128^

*NRCM* neonatal rat cardiomyocytes, *ARCM* adult rat cardiomyocytes, *ARVMs* adult rat ventricular myocytes, *3-MA* 3-methyl adenine, *BafA1* bafilomycin 1, *AMCM* adult mice cardiomyocytes, *TFEB* transcription factor EB, *Nrf-2* Nuclear factor erythroid 2-related factor 2, *LC3-II* microtubule-associated protein 1A/1B-light chain 3, *Atg* autophagy-related protein, *CQ* chloroquine, *SIRT1* Sirtuin 1, *DIC* doxorubicin-induced cardiotoxicity, *MEFs* mouse embryonic fibroblasts, *SD* Sprague Dawley, *sh* short hairpin RNA; ↓ decrease, ↑ increase.

**Fig. 1:**
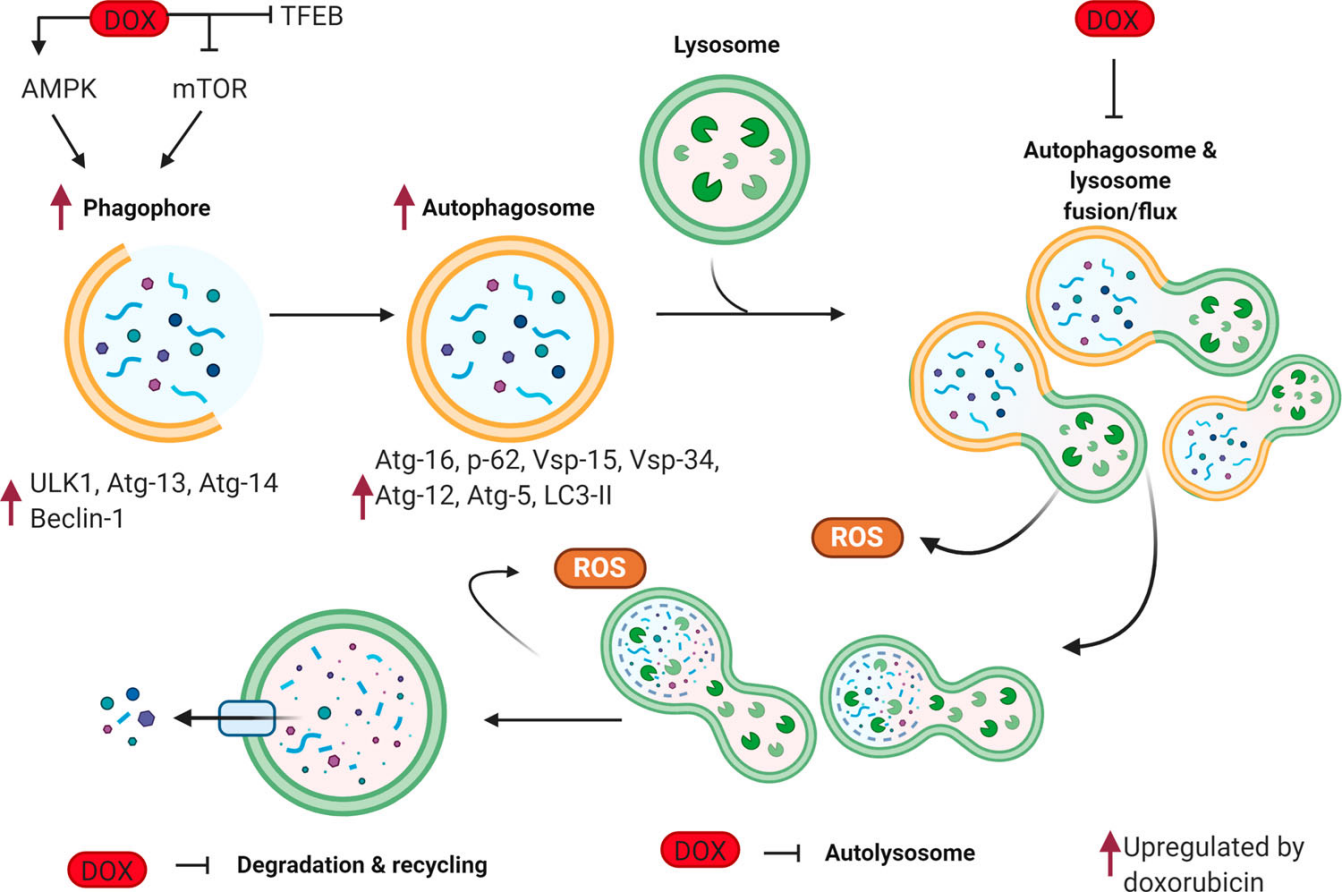
Role of doxorubicin in autophagy-related cardiomyocyte death. Schematic representation of autophagy during doxorubicin treatment. Doxorubicin disrupts autophagy by: inducing initiation through AMPK activation and/or mTOR inhibition, the formation of the phagophore, upregulation of Atg proteins, and by blocking lysosomal proteolysis resulting in accumulation of autophagosomes and autolysosomes and reactive oxygen species. DOX doxorubicin, AMPK 5′ AMP-activated protein kinase, mTOR mammalian target of rapamycin, TFEB transcription factor EB, Atg autophagy-related protein, LC3II microtubule-associated protein 1A/1B-light chain 3, ROS reactive oxygen species.

Recent evidence has helped to resolve this apparent discrepancy by proposing that doxorubicin initially induces autophagy but then blocks it^30–33^ resulting in the accumulation of undegraded autophagosomes and autolysosomes which exacerbate the damage in cardiomyocytes leading to their death (Fig. 1). Specifically, a low dose of doxorubicin resulted in LC3-II, p62, and Beclin1 protein expression increase, indicating induction of autophagy^30^. However, when assessing downstream activities of autophagy, doxorubicin impaired the autophagic flux and inhibited lysosomal acidification in cardiomyocytes. This blockade in the autophagic process resulted in the accumulation of undegraded autolysosomes, which in turn leads to ROS production and DIC^30^ (Fig. 1). Mice haploinsufficient for Beclin 1 and therefore with reduced autophagy initiation capacity, had a reduced number of unprocessed autolysosomes compared to the wildtype mice upon doxorubicin treatment, and consequently, this lead to a decreased in ROS production and attenuation of DIC. Conversely, increasing the level of autophagy by Beclin 1 overexpression augments DIC^30^. Similarly, doxorubicin inhibits transcription factor EB (TFEB) expression which in turn suppresses lysosomal proteolysis resulting in autolysosome accumulation and reduced viability^33^. TFEB is a positive regulator of autophagy critically involved in autophagosomal processing and lysosomal integrity, function, and fusion^33^. Genetic restoration and pharmacologic activation of TFEB using Torin-1 prevent doxorubicin-induced inhibition of cathepsin B a lysosomal cysteine protease and ROS production, resulting in increased cell viability^33^. In summary, recent studies demonstrate that doxorubicin suppresses lysosomal proteolysis resulting in autophagosome and autolysosome accumulation promoting ROS production and cell death. In consequence, blocking autophagy initiation or stimulating lysosomal function serves as a potential therapeutic approach as it reduces the accumulation of autolysosomes^13,30,31,33^ and attenuates ROS production.

These findings may explain in part the discrepant results obtained in assessing the effect of doxorubicin in autophagy. Doxorubicin has an opposite effect on early (autophagy induction) vs. later stages of autophagy (autophagy blockade)^30–33^ and as a result, assessing doxorubicin’s effect on autophagy could generate a different outcome depending on which stage of autophagy is being evaluated. Differences in experimental settings including in vitro vs. in vivo models, different doxorubicin doses, different duration of treatments, as well as cross-sectional vs. longitudinal measurements could also give rise to the discrepancies seen in the literature^13,30^.

On the other hand, research examining the effect of autophagy *prior* to doxorubicin treatment is in agreement and shows protection from DIC^34–38^. Stimulating autophagy prior to doxorubicin treatment by mTOR inhibition via rapamycin treatment or by caloric restriction results in improved cardiac outcomes and reduced lethality in rodents models^34–38^. A possible explanation for this is that induction of autophagy prior to doxorubicin treatment may render cells better able to tolerate the cellular stress of doxorubicin by eliminating damaged cellular components.

## Ferroptosis

**Fig. 2:**
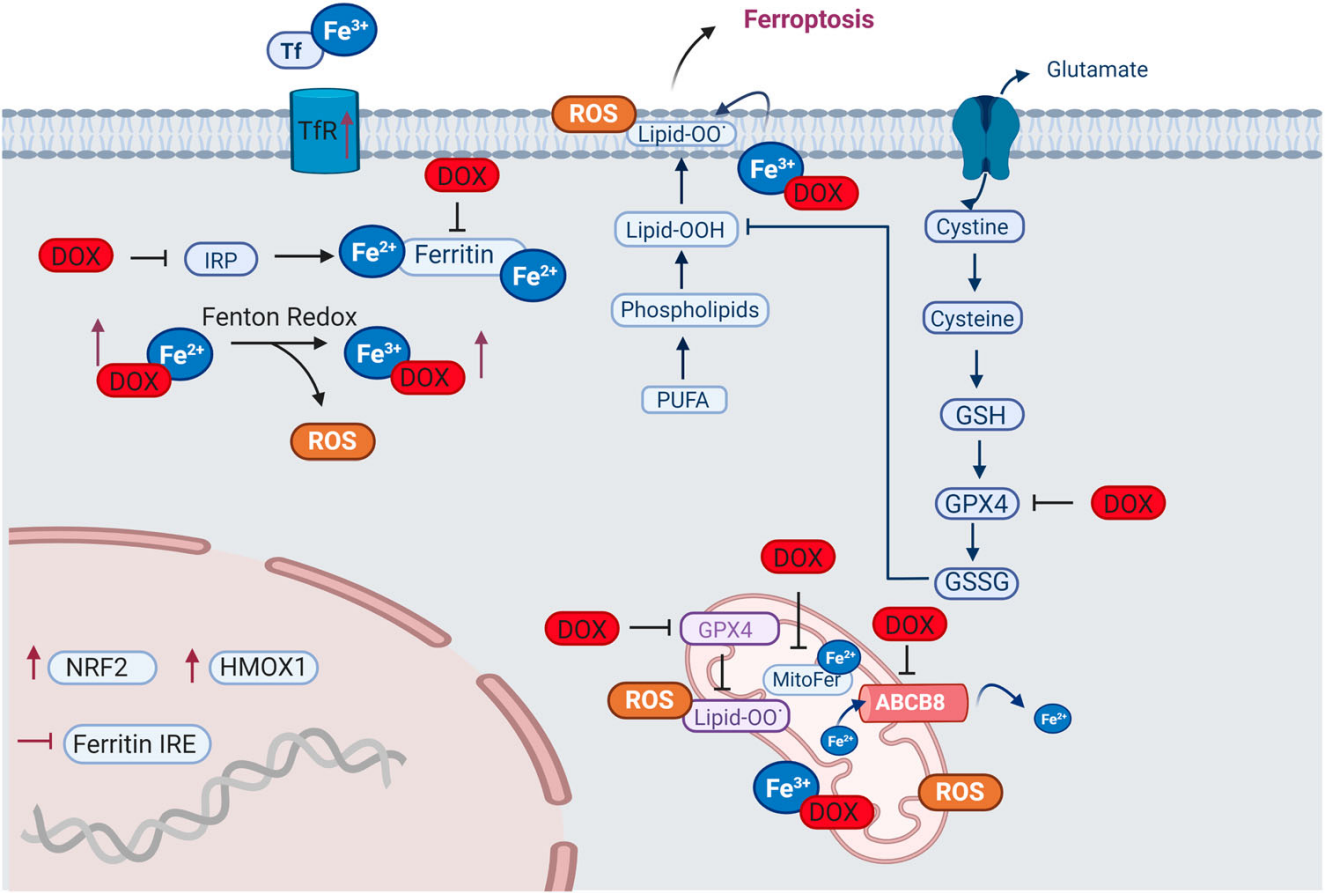
Doxorubicin-induced ferroptosis. Schematic representation of doxorubicin-induced ferroptosis pathway. Doxorubicin treatment results in iron overload through upregulation of TfR and inactivation of ferritin. Free iron complexes with doxorubicin and through the Fenton reaction create reactive oxygen species (ROS). Doxorubicin induces lipid peroxidation by inhibiting cytosolic and mitochondrial GPX4 resulting in ferroptosis. In the mitochondria, doxorubicin causes iron overload by blocking MitoFer and ABCB8. In the nucleus, activation of NRF2 results in upregulation of HMOX1 leading to heme degradation and resulting in excess free iron and ferroptosis. Tf transferrin, TfR transferrin receptor, IRP iron response regulatory protein, IRE Iron response element, NRF2 nuclear factor erythroid 2-related factor, HMOX1 heme oxygenase 1, PUFA polyunsaturated fatty acids, Lipid-OO lipid peroxides, GSH reduced glutathione, GPX4 glutathione peroxidase, GSSG glutathione disulfide, H_2_O_2_ oxygen peroxide, MitoFer mitochondria ferritin. ABCB8 ATP-binding cassette sub-family B member 8, ROS reactive oxygen species.

Ferroptosis, another pathway by which doxorubicin exerts its cardiotoxic effect, is an RCD pathway characterized by the iron-dependent accumulation of lipid peroxides^39^. Lipid peroxides are one of the many sources of ROS involved in DIC and the role of iron in DIC is been well characterized^6,40,41^ (Fig. 2). Doxorubicin treatment increases the labile iron pool in cells which is toxic^40^. In line with this, rats fed an iron-rich diet and treated with doxorubicin have worse cardiac outcomes than rats fed a control diet^42^. Doxorubicin and its metabolites can affect iron homeostasis by inactivating iron regulatory proteins 1 and 2 (IRP1 and IRP2)^41^. Inactive IRPs bind to iron-response elements (IREs) modifying the expression of genes involved in iron metabolism^41^. Doxorubicin disrupts the mRNA of ferritin’s IRE, leading to reduced ferritin and increased labile iron^43^. Similarly, doxorubicin upregulates TfR allowing more iron to enter the cell leading to excess free intracellular iron^44^. Conversely, inhibition of TfR with an anti-TfR antibody reduces iron uptake and leads to reduced intracellular oxidant formation and cell death^44^. In addition, humans with mutations in the human hemochromatosis protein (HFE)—which controls the interaction of TfR with transferrin—experience iron overload in different tissues including the heart. Based on this, mutations in the HFE gene were hypothesized to make patients more susceptible to DIC and two variants in the HFE gene have been associated with increased susceptibility to DIC; a finding not replicated in all studies^45–48^. Deletion of the HFE protein in mice results in excess free iron and increased susceptibility to DIC^49^. Finally, the importance of iron overload in the heart during doxorubicin treatment is highlighted by the clinical use of the iron chelator dexrazoxane, the only clinically approved cardioprotection.

Mitochondrial iron overload is a central underlying mechanism of DIC. Heart biopsy specimens from patients with heart failure due to DIC show excess iron in their mitochondria compared to the hearts from other non-DIC heart failure patients or healthy individuals^50^. Excess iron in the mitochondrial could be explained by doxorubicin-induced downregulation of ABCB8 protein that controls iron export from the mitochondria^50^. Another protein that is important in mitochondria iron homeostasis is mitochondrial ferritin (MitoFer), which, similar to its cytoplasmic equivalent, stores free iron^51^. Genetic inactivation of MitoFer in rodents results in increased DIC^51^.

Recent understanding of ferroptosis helps to explain in part the long-recognized role of abnormal iron homeostasis in DIC and provides new targets for preventative therapies (Fig. 2). Mice administered doxorubicin and ferrostatin-1, a ferroptosis inhibitor, have improved survival^52,53^. The effect of ferrostatin-1 appears to be superior to agents that inhibit other RCD pathways including apoptosis and necroptosis^52,54^. Doxorubicin triggers ferroptosis in mice via the activation of nuclear factor erythroid 2-related factor 2 (*nrf-2*) which leads to the upregulation of heme oxygenase 1 (*hmox1*)^52^. Hmox1 catalyzes heme degradation inducing the release of free iron and ultimately leading to the accumulation of oxidized lipids in the mitochondria membrane. In addition, doxorubicin downregulates a key anti-ferroptotic protein, glutathione peroxidase 4 (GPX4) in the cytosol and mitochondria and induces excessive lipid peroxidation through doxorubicin–Fe^2+^ complex in the mitochondria^54^. These findings highlight the importance of ferroptotic death in DIC^52–54^ and the crucial role that mitochondria play in doxorubicin-induced ferroptosis^52,54^. Agents such as MitoTempo, a mitochondrial anti-oxidant that completely blocked ferroptosis^52^ and ferrostatin-1 could be promising cardioprotectants to blunt the cardiotoxic effects of doxorubicin^52–54^.

## Necroptosis

**Fig. 3:**
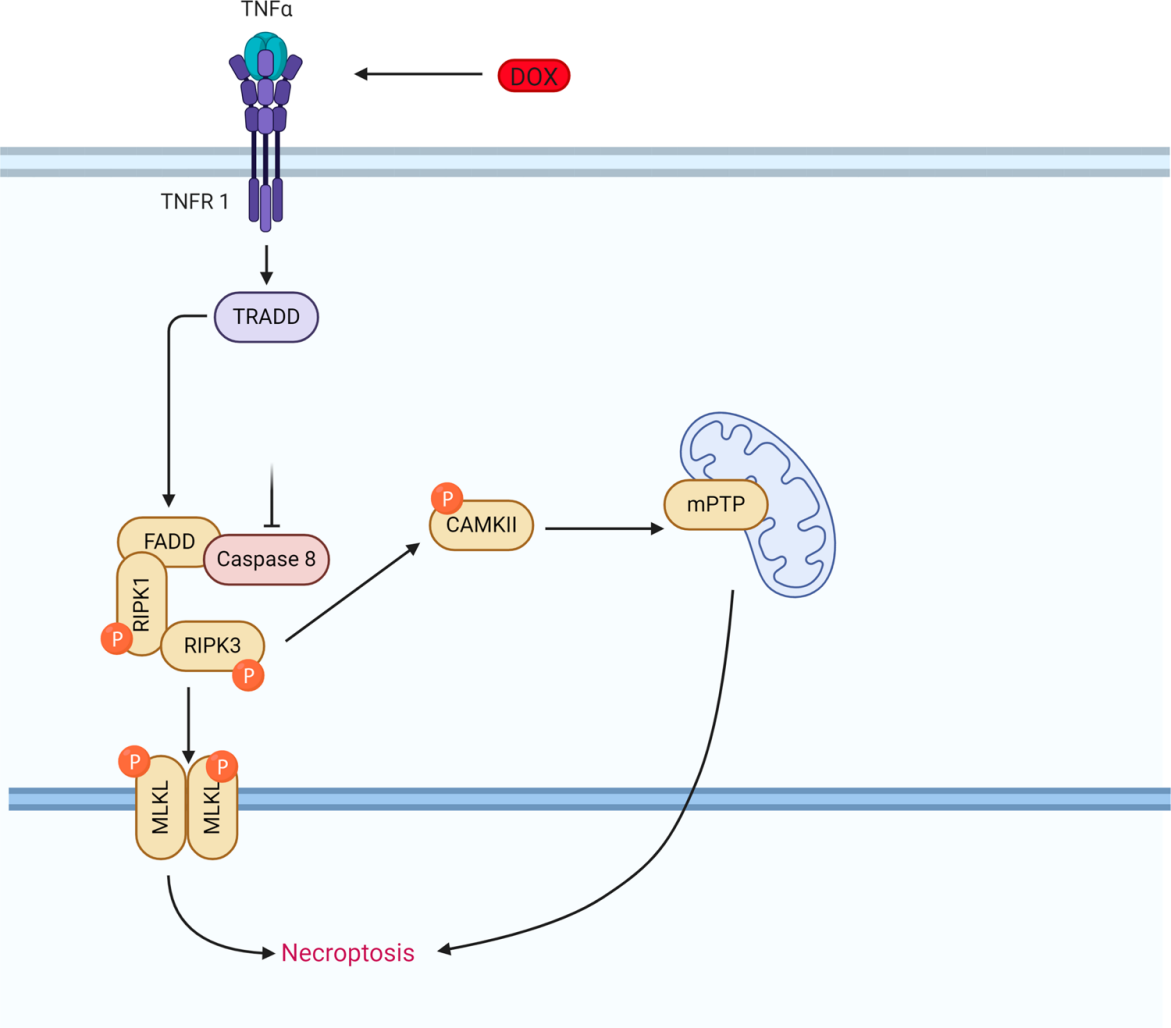
Doxorubicin-induced necroptosis. Schematic representation of the doxorubicin-induced necroptosis pathway. Doxorubicin causes upregulation of TNFα, activating TRADD and FADD, and upon caspase 8 inhibition and activation of RIPK1, RIPK3, and MLKL induces cell death via necroptosis. Doxorubicin can also activate necroptosis via the RIPK1 independent pathway, where RIPK3 activates CAMKII and mitochondrial permeability transition pore (mPTP) resulting in membrane potential and integrity loss. TNFα tumor necrosis factor-alpha, TRADD tumor necrosis factor receptor type 1 associated death domain protein, FADD Fas-associated protein with death domain, RIPK receptor-interacting serine/threonine-protein kinase, CAMKII calcium/calmodulin-dependent protein kinase II association domain, MLKL mixed lineage kinase domain-like, mPTP mitochondrial permeability transition pore.

Doxorubicin also activates another form of cell death, necroptosis (Fig. 3). Necroptosis is a regulated form of necrosis that involves the release of death-signaling cytokines^55,56^. Mechanistically, tumor necrosis factor-α (TNF-α) activates the protein TNFR-associated death protein (TRADD) through TRFR1 and phosphorylates receptor-interacting serine/threonine-protein kinase 1(RIPK1) which recruits and phosphorylates RIPK3 forming the necroptosome^55,56^. The necroptosome subsequently phosphorylates the mixed lineage kinase domain-like protein (MLK1) which ruptures the plasma membrane and allows the release of organelles and inflammatory factors inducing an immune response and leading to cell demise^55^.

Left ventricular samples from end-stage heart failure patients have increased expression of necroptotic proteins, suggesting a role for necrotptosis in heart failure^57^. In DIC specifically, necrostatin-1 has a protective effect in vitro, while dexrazoxane during doxorubicin treatment is both able to downregulate apoptosis and necroptosis suggesting the involvement of both pathways in the pathogenesis of DIC^58^. In addition, doxorubicin activates necroptosis through an alternative and novel necroptotic pathway, which together with necrosis causes more cell death than apoptosis (Fig. 3)^59^. Doxorubicin upregulates RIPK3 which binds and phosphorylates calmodulin kinase II (CaMKII) which in turn regulates the opening of the mitochondrial permeability transition pore (mPTP) leading to necroptosis and apoptosis. Evidently, necroptosis can occur in the absence of RIPK1 and MLKL^59^. The characterization of the exact players in doxorubicin-induced necroptosis will allow the identification of novel agents that may block this pathway. To date, necrostatin-1 and KN-93, a CAMKII inhibitor, have shown protection from DIC in experimental models^58,59^.

## Pyroptosis

Pyroptosis was first demonstrated in 2001^60^ and is now widely recognized to play a crucial role in the pathogenesis of cardiovascular diseases^61^. Pyroptosis is characterized by increased inflammation and activation of caspase -1, caspase-3, caspase-4, and caspase-11 as well as NLR family pyrin domain containing 3 (NLRP3) leading to the cleavage of Gasdermin D (GSDMD) or GSDME and to the rupture of the plasma membrane that allows the release of interleukin-1beta (IL-1β) and IL-18^62–64^.

**Fig. 4:**
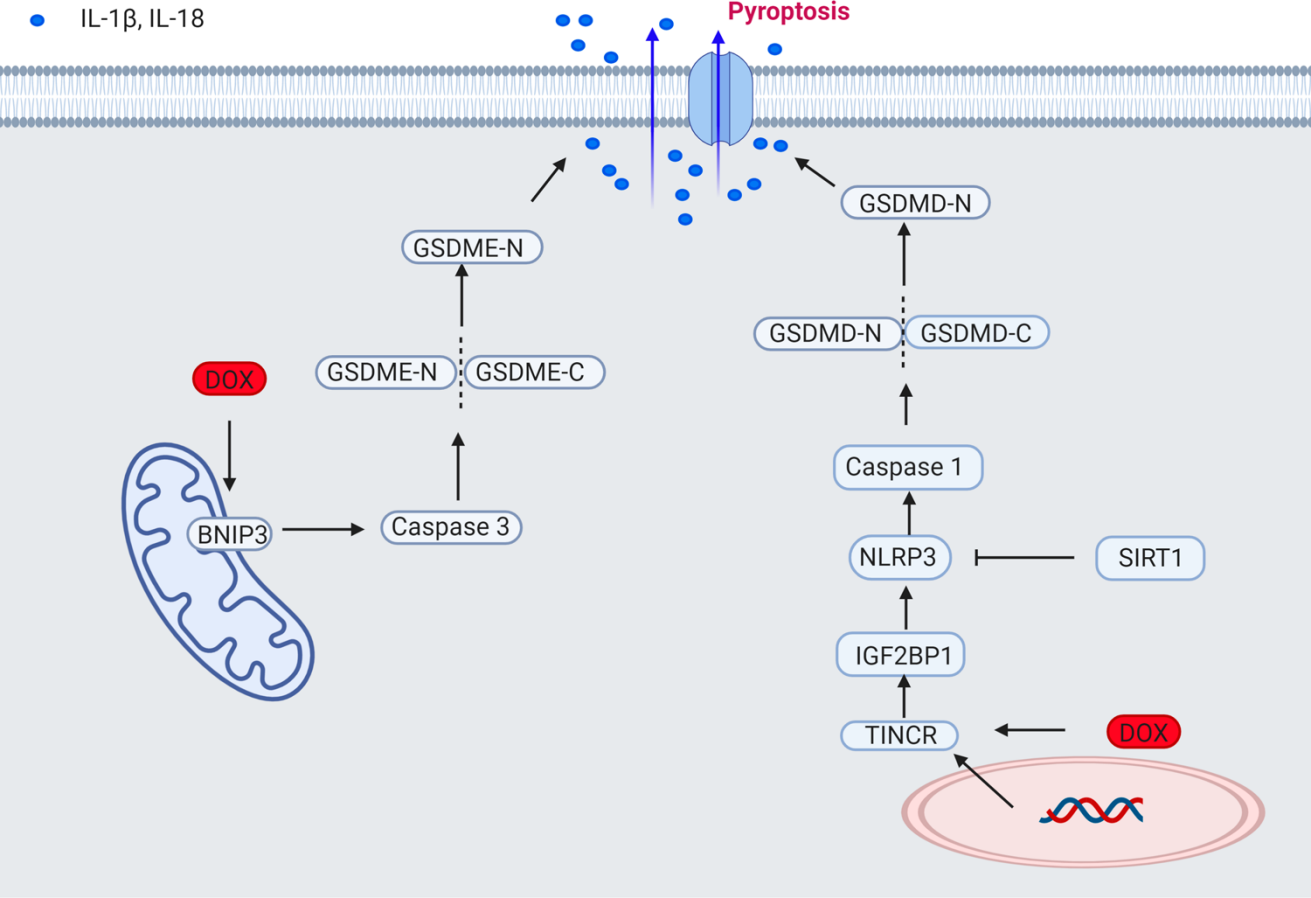
Doxorubicin-induced pyroptosis. Schematic representation of doxorubicin-induced pyroptosis in the heart. Doxorubicin induces pyroptosis via the upregulation of TINCR, which recruits IGF2BP and increases the expression of NLRP3 leading to activation of caspase-1, the cleavage of GMDSD-N and the release of IL-1β, IL-18. Pyroptosis is also induced via BNIP3 activation in the mitochondria, which activates caspase 3 and causes GSDME-dependent pyroptosis. Sirtuin 1 activation inhibits NLRP3 and protects cardiomyocytes from doxorubicin-induced pyroptosis. BNIP3 BCL2 interacting protein 3, GSDMD gasdermin D, GSDME gasdermin E, TINCR terminal differentiation-induced NcRNA, IGF2BP1 insulin-like growth factor 2 mRNA-binding protein 1, NLRP3 NOD−, LRR−, and pyrin domain-containing protein 3, IL interleukin.

Doxorubicin-induced pyroptosis proceeds via the upregulation of Terminal Differentiation-Induced Non-Coding RNA (TINCR), which recruits IGF2BP and increases the expression of NLRP3 leading to activation of caspase-1, the cleavage of GMDSD-N, and the release of IL-1β, IL-18^65^ (Fig. 4). The inhibition of NLRP3 using MCC950 protected the cells from doxorubicin-induced cell death^65^. A different pathway by which doxorubicin triggers pyroptosis that acts through the activation of Bnip3 in the mitochondria has also been demonstrated^66^ (Fig. 4). Doxorubicin increases the expression of BH3-only protein Bcl-2/adenovirus E1B 19-kDa-interacting protein 3 (Bnip3) which in turn activates caspase 3 and causes GSDME-dependent pyroptosis. Disruption of GSDME and silencing of Bnip3 protects cardiomyocytes from DIC in vitro^66^. Protection from DIC has also been shown to occur through the NLRP3/caspase 1signalling blockade using embryonic stem cell-derived exosomes, overexpression of heat shock protein 22, and pharmacologic inhibition of NLRP3^67–69^. Sirtuin 1 activation inhibits NLRP3 and protects cardiomyocytes from doxorubicin-induced pyroptosis^70^. Inhibition of pyroptosis-related molecules, such as NLRP3, caspase 1, Bnip3, may represent a strategy to limit DIC.

## Apoptosis

**Fig. 5:**
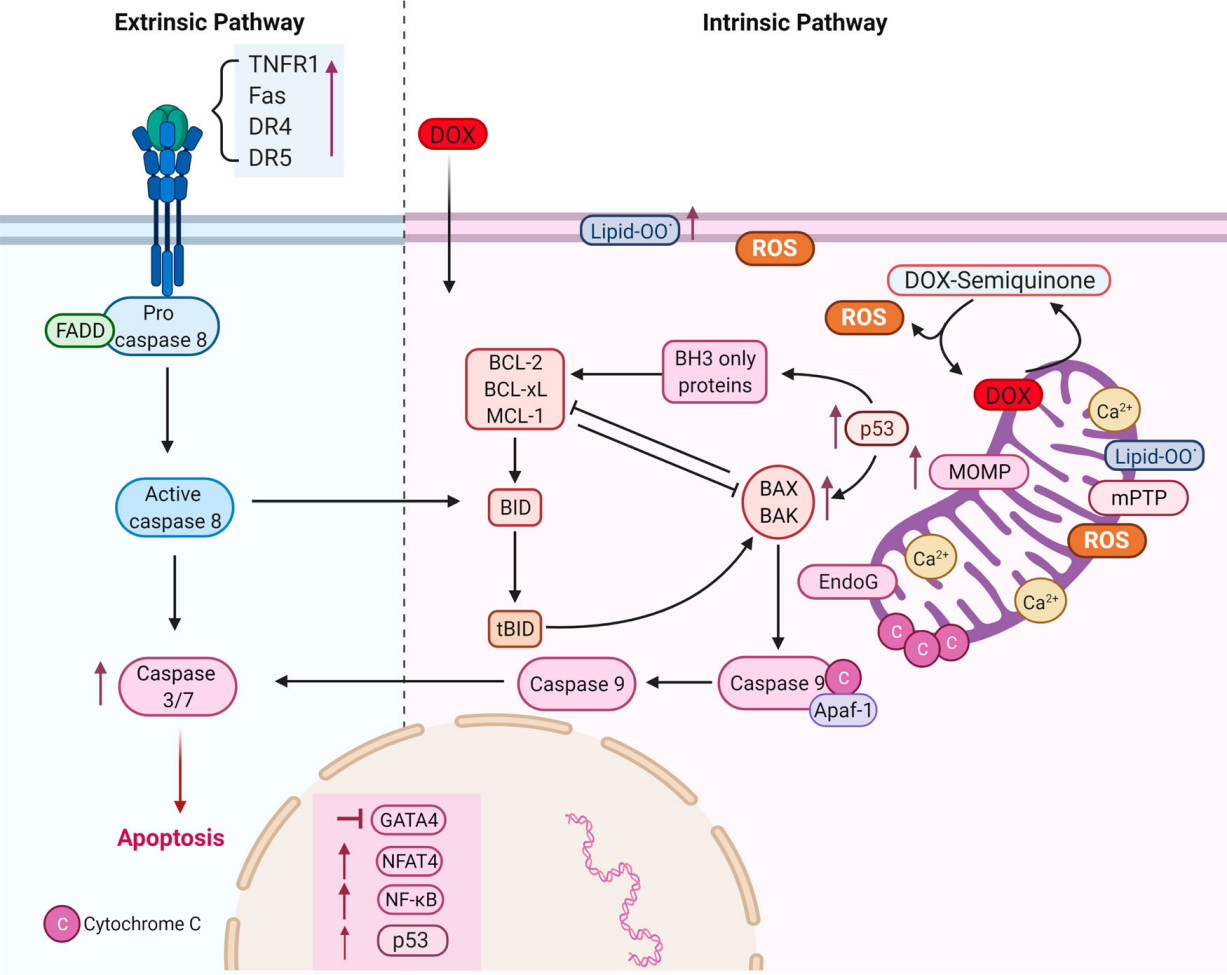
Doxorubicin-induced apoptosis. Schematic representation of doxorubicin-induced apoptosis in the heart. Doxorubicin-induced upregulation of p53, Bax/Bak, and downregulation of GATA4 and Bcl-XL activating caspases 9, 3, and 7 resulting in apoptotic death. Mitochondrial calcium overload and activation of mitochondrial permeability transition pore (mPTP) lead to mitochondrial membrane potential loss, mitochondrial swelling, and outer membrane rupture allowing the release of endonuclease G (EndoG), cytochrome c and activation of caspase 9. Doxorubicin induces the extrinsic apoptotic pathway via the upregulation of death receptors and the activation of NFAT and NF-κΒ. DR: death receptor, TNFR1 tumor necrosis factor receptor 1, FADD Fas-associated protein with death domain, DR death receptor, lipid-OO lipid peroxides, EndoG endonuclease G, Ca2+ calcium, ROS reactive oxygen species, mPTP mitochondrial permeability transition pore, MOMP mitochondria outer membrane permeability, DOX doxorubicin, Bax Bcl-2-associated X protein, Apf-1 apoptosis protease factor-1, BID BH3 interacting-domain death, NF-κΒ nuclear factor-κB, NFAT4 nuclear factor of activated T-cells, GATA4 GATA-binding protein 4.

The apoptotic pathway is the most well-characterized programmed cell death pathway and is the most studied in DIC. In summary, doxorubicin treatment causes excess oxidative stress (lipid peroxidates, doxorubicin’s reduced semiquinone moiety, charged doxorubicin iron complex, respiratory chain failure, peroxynitrites, and others) and mitochondrial damage which triggers cell death pathways including apoptosis^6,71–73^. These events activate the intrinsic pathway and together with mitochondrial calcium overload induce the mPTP, resulting in mitochondrial membrane potential loss, mitochondrial swelling, and outer membrane rupture which eventually leads to the release of endonuclease G (EndoG), cytochrome c, and apoptosis-inducing factor (AIF) in the cytosol^72,74^. Once in the cytosol, cytochrome c complexes with the adapter protein Apaf-1, dATP, and caspase 9 forming the apoptosome. Apoptosome formation leads to the cleavage and activation of caspase 9, which in turn cleaves and activates caspase 3 resulting in cell death. Doxorubicin can activate the intrinsic apoptosis pathway via Bax/Bak activation and translocation from the cytosol to the outer membrane of mitochondria inducing the mitochondrial outer membrane permeabilization, allowing the diffusion of several proteins to the cytoplasm including strong pro-apoptosis factor cytochrome c^75^. Doxorubicin activates intrinsic apoptosis by a number of mechanisms, including upregulation of p53 which leads to Bax upregulation^76,77^, downregulation of GATA4 which decreases antiapoptotic Bcl-XL expression^78^, activation of JNK and MAPK, and inactivation PI-3K/Akt pro-survival pathway^41,72,79–82^ (Fig. 5).

Doxorubicin also induces the extrinsic apoptotic pathway in cardiomyocytes^83^. Death ligands, such as FasL and TNFα, bind to their receptors and trigger the recruitment of cytosolic proteins Fas-associated via death domain (FADD) and TNFR-associated death domain (TRADD)^72,84^. FADD and TRADD recruit caspase 8 and activated caspase 8 can activate caspase 3 which results in apoptosis^85^. Doxorubicin activates the extrinsic apoptotic pathway by activation of nuclear factor-activated T cell-4 (NFAT4) and NF-κB, leading to the upregulation of Fas/FasL and p53; and, downregulation of FLIP, a FLICE/caspase-8 inhibitory protein, which induces Fas-mediated cell death^41,84,86,87^ (Fig. 5).

Recent evidence suggests that circulating death ligands can act as clinical biomarkers and understanding the extrinsic pathway could have a great therapeutic potential. In a study, investigating the effect of doxorubicin on death ligands in induced pluripotent stem cell derived cardiomyocytes (iPSC-CMs), death receptors TNFR1, Fas, and death receptor 5 (DR5) were upregulated and DIC was enhanced by physiologically relevant TNF-related apoptosis inducing ligand (TRAIL)^88^. This finding suggests that elevated serum levels of specific TNF cytokines due to certain diseases and treatment conditions, could be predictive for the risk of cardiotoxicity prior to the administration of doxorubicin to a patient^88^. Another study using the same model of iPSC-CMs showed that FAS, DR4, and DR5 are the most significantly upregulated genes in RNA-seq data and found that the extrinsic pathway is more upregulated than the intrinsic apoptotic pathway suggesting that blocking the extrinsic pathway could be a more beneficial approach in treating DIC^89^.

p53 is a key modulator of cell death and has long been implicated with DIC. Doxorubicin upregulates p53 which turns on signaling pathways resulting in extrinsic and intrinsic apoptosis^72,89^. However, recent evidence suggests that p53 involvement in DIC is more complex than originally thought. p53 displays both protective and deleterious effects on the cardiomyocyte depending on the time of doxorubicin treatment and the dose. In the acute setting p53 blockade attenuates apoptotic death, however, chronic p53 deletion augments mitochondrial toxicity and cell death^90,91^. p53 has dose-specific effects as well whereby high doxorubicin doses induce p53 dependent apoptosis, while with lower doxorubicin concentrations the mitochondria bioenergetic failure is the main cardiotoxic event^92^.

Another recent finding is the involvement of Wnt signaling in DIC^93–96^. Wnt/β-catenin signaling inhibits apoptosis and has a protective effect in DIC with doxorubicin downregulating this pathway^93,95,97^. The Wnt/β-catenin signaling pathway is crucial for many developmental processes including heart development and homeostatic processes, such as apoptosis, cell proliferation, migration, and differentiation^98,99^. Deregulation of Wnt/β-catenin is associated with a variety of cardiovascular diseases and impaired cardiogenesis^100–102^.

A protein involved in the Wnt signaling, the secreted frizzled-related protein 1 (sFRP1), has a location-dependent effect in response to DIC^93^. Doxorubicin increases extracellular secretion of sFRP1 and depletes intracellular sFRP1 concentration^93^. Clinically, sFRP1 expression is upregulated in samples of DIC hearts compared with healthy hearts, a finding that was replicated in experimental in vivo models^93^. Inhibition of sFRP1 secretion attenuated DIC in vitro via the activation of Wnt/β-catenin signaling, while increasing the secretion of sFRP1 had the opposite effect. However, the intracellular concentration of sFRP1 was significantly reduced after doxorubicin exposure both in vitro and in vivo. Knockdown of sFRP1 increased sensitivity to DIC, while overexpression of sFRP1 protected the hearts from DIC. In summary, sFRP1 has a protective intracellular effect but when secreted it damages the heart. As a result, the oversecretion of sFRP1 can be used as a clinical biomarker for DIC. As sFRP1 can regulate both the canonical Wnt/β-catenin and noncanonical Wnt/PCP-JNK pathways the same group also investigated the effect of doxorubicin on Wnt/PCP-JNK signaling^103,104^. Although, doxorubicin downregulates Wnt/β-catenin signaling, in the case of Wnt/PCP-JNK signaling it upregulates it^94^. Pretreatment with a Wnt/PCP-JNK signaling inhibitor, reduced doxorubicin-induced apoptosis in vitro, while sFRP1 overexpression protected from DIC through the downregulation of Wnt/PCP-JNK signaling^94^. Finally, another group reported that Dickkopf-1 (Dkk1) gets activated during doxorubicin treatment and inhibits the canonical Wnt/β-catenin pathway leading to increased cardiomyocyte apoptosis and mitochondria dysfunction^95^. Blockade of Dkk1 attenuated DIC, while overexpression of Dkk1 had the opposite effect. In summary, activating the Wnt/β-catenin signaling pathway during doxorubicin treatment can protect from DIC according to in vivo and in vitro models, and pharmacological agents blocking inhibitors of this pathway such as Dkk1 or activating inducers such as intracellular sFRP1 can protect from DIC.

### Sirtuins in DIC

In the heart, sirtuins (SIRT) 1, 2, 3, and 6 activate the autophagic process, repress apoptosis and protect the heart from various cardiovascular threats such as atherosclerosis, ischemia-reperfusion injury, myocardial hypertrophy, diabetic cardiomyopathy, and cardiac hypertrophy^105,106^.

**Fig. 6:**
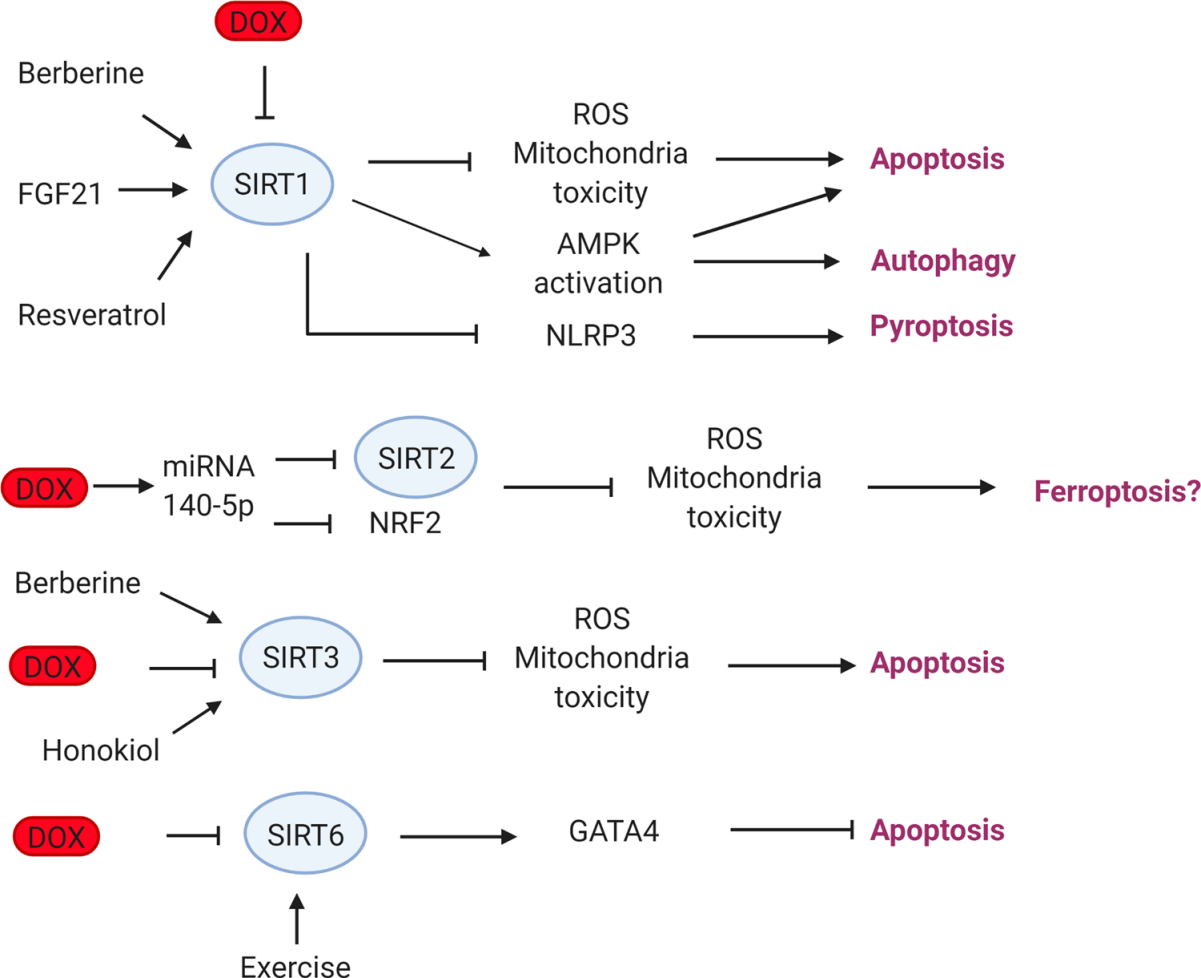
Role of Sirtuins in doxorubicin-induced cardiotoxicity. Doxorubicin downregulates SIRT1, causing increased oxidative damage, loss of mitochondria integrity, AMPK, and NLRP activation resulting in increased apoptosis, autophagy, and pyroptosis. Berberine, FGF21, and resveratrol protect from DIC via SIRT1 activation. Doxorubicin downregulates SIRT2 via miRNA-140-5p, leading to reduced SIRT2 and NRF2 expression. SIRT2 has been implicated with iron homeostasis while NRF2 activation is involved in doxorubicin-induced ferroptosis. Doxorubicin-induced downregulation of SIRT3 causes increased oxidative damage and loss of mitochondria integrity leading to increased apoptosis. Berberine and honokiol protect from DIC via SIRT3 upregulation. Doxorubicin treatment reduces the expression of SIRT6 leading to repression of GATA4 and increased apoptosis. SIRT Sirtuin, DOX doxorubicin, FGF21 fibroblast growth factor 21, Nrf2 nuclear factor erythroid 2-related factor 2, GATA4 GATA-binding protein 4.

Sirtuins may play a protective role in DIC (Fig. 6). In rodents, doxorubicin treatment downregulates Sirt1 expression, increases ROS production and cell death. Overexpression of Sirt1 or resveratrol treatment, a SIRT1 agonist, and an autophagy activator, reverses the DIC phenotype^107^. Similarly, doxorubicin reduces Sirt1 expression in the myocardium, leading to excess cellular damage, mitochondrial dysfunction, oxidative stress, and apoptosis. Berberine, an alkaloid herbal extract, and agonist of Sirt1 reduces DIC^27^. The effect of Sirt1 on DIC appears to be dependent on fibroblast growth factor 21 (FGF21), as inhibition of Sirt1 attenuates the protective effects of FGF21 against DIC in mice^108^.

SIRT3, a mitochondrial sirtuin protects the heart against myocardial hypertrophy, diabetic cardiomyopathy, and DIC. Doxorubicin treatment reduces Sirt3 expression in vivo and in vitro in rodent hearts^26,109,110^. In the same manner, overexpression of Sirt3 protects the heart from doxorubicin by reducing oxidative damage and maintains mitochondrial integrity^26,109,111^. Sirt3 activators, honokiol, and berberine improve cellular damage in the heart induced by doxorubicin^26,110^. Consistent with this, *Sirt3* knockout mice died before completing doxorubicin treatment^109^ (Fig. 6).

SIRT6 is located predominately in the nucleus and similarly to SIRT3 protects the heart from cardiac hypertrophy and DIC^105^. Doxorubicin treatment reduces the expression of SIRT6 leading to repression of GATA4 an antiapoptotic gene^112^. Under normal conditions, SIRT6 recruits TIP60 acetyltransferase to acetylate GATA4, and then GATA4 suppresses SIRT6’s deacetylase activity. Doxorubicin can block the assembly of the SIRT6–TIP60–GATA4 trimeric complex and decrease the expression of GATA4 which results in cell death. In addition, GATA4 hyperacetylation-mimic protects against DIC providing evidence that the Sirt6–TIP60–GATA4 trimeric complex could be a new therapeutic target against DIC^112^. Finally, a recent study showed that pregnant mice who exercised during doxorubicin treatment managed to increase their Sirt6 expression and improve viability in progeny’s hearts compared to the control group that did not exercise^113^ (Fig. 6).

The role of SIRT4, 5, and 7 in DIC has yet to be studied. One study has investigated the effect of SIRT2 on DIC and showed that doxorubicin treatment increases expression of the miRNA 140-5p, which in turn suppresses the expression of Nrf2 and Sirt2 leading to increased cell death^114^.

In summary, in vivo, and in vitro models provide mounting evidence that activating sirtuins with agents such as berberine and resveratrol reduces the severity of DIC and may serve as potential therapeutic approaches.

## Discussion

As the number of cancer survivors is growing, chemotherapy-related cardiotoxicity represents an increasing health problem for the future. Doxorubicin causes cardiotoxicity through multiple mechanisms including autophagy, apoptosis, necroptosis, ferroptosis, pyroptosis, and others. Understanding these mechanisms is essential in order to identify pharmacological agents that block these pathways and also understand the functional impact of genetic variants that have been associated with DIC.

Substantial effort has been put in utilizing agents that decrease ROS production, however, they have failed to reduce cardiotoxicity, indicating that the mechanism of DIC involves more than just oxidative stress as was initially suggested^115,116^. With a clearer understanding of the predominant cell death pathways by which doxorubicin kills the cell, new agents that target these pathways can be trialed. For example, triggering autophagy prior to doxorubicin treatment using pharmacological agents or caloric restriction has promising results in attenuating DIC in rodent models^34–38^. Similarly, blockade of ferroptosis shows encouraging outcomes in pre-clinical models^52,54^. However, these novel findings are yet to be assessed in clinical trials.

Many cardioprotectants discovered that protect from DIC in experimental models fail to reach clinical trials. One reason is that there are controversies and discrepancies in the RCD pathways involved in DIC. For example, it remains unclear whether autophagy plays a primarily protective or disruptive effect during doxorubicin treatment. Given the complexity of doxorubicin’s cellular effects, understanding the precise cell death pathways involved remains a significant challenge.

One promising opportunity for future study is the effect of genetic variants associated with DIC that may offer mechanistic insights into cell death pathways involved in DIC. For example, a genetic-wide association study identified a genetic variant in the retinoic acid receptor gamma (RARG-S427L) that increases susceptibility to doxorubicin^117^. Recently, the functional impact of this variant was validated in an iPSC-CMs model^118^. Doxorubicin-treated iPSC-CMs harboring this mutation experienced increased cell death, ROS production, and double-strand breaks^118^. The precise mechanism by which variation in the RARG causes cell death is yet to be elucidated. Two possible mechanisms could explain how this genetic variation affects doxorubicin-induced cardiomyocyte death (i) RARG-S427L upregulates TOP2B leading to double-strand breaks^117,118^, (ii) RARG-S427L could make cells more susceptible to doxorubicin via necroptosis; as it has been shown that RARG can complex to RIPK1 to induce necroptosis in mouse embryonic fibroblasts in response to chemotherapy drugs including doxorubicin^119^. Verifying genetic variants that are associated with DIC and understanding their mechanisms not only will it allow to identify their impact on cell signaling pathways but it will also have a tremendous therapeutic potential allowing genetic testing prior to the administration of the drug.

In addition, deepening our understanding of the RCD pathways involved in cardiomyocyte cell death may improve the evaluation of drug-induced cardiotoxicity. Many studies evaluate cardiotoxicity based primarily on the apoptotic pathway. However, other RCD pathways may have a significant contribution to drug-related cardiomyocyte toxicity and therefore a more comprehensive experimental approach may better predict clinical cardiotoxicity.

**Fig. 7:**
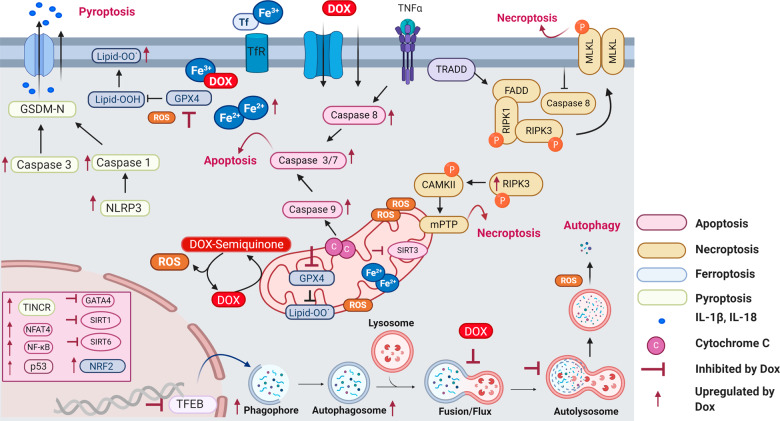
Doxorubicin-induced regulated cell death pathways. Summary of regulated cell death pathways triggered by doxorubicin in the heart. Doxorubicin triggers ROS production by inducing initiation of autophagy and by blocking lysosomal proteolysis resulting in the accumulation of autophagosomes and autolysosomes. Doxorubicin undergoes both redox cycling forming dox-semiquinone moieties and Fenton reaction creating oxidative species. Excess iron and lipid peroxidation due to doxorubicin treatment results in ferroptosis. Doxorubicin activates NLRP inducing the release of Il-1β and Il-18 resulting in death due to pyroptosis. RIPK1 and RIPK3 activation due to doxorubicin treatment leads to phosphorylation of MLKL and necroptosis. Doxorubicin causes necroptosis through RIPK1 independent pathway by activating RIPK3 and CAMKII leading to mPTP and membrane potential loss. ROS trigger p53 activation and GATA4 downregulation stimulating the intrinsic apoptotic pathway. Doxorubicin treatment upregulates death receptors and together with the activation of NFAT and NF-κΒ the extrinsic apoptotic pathway is triggered. Pink color capsules represent apoptosis, brown color capsules represent necroptosis, blue color capsules represent ferroptosis and white capsules represent pyroptosis. DOX doxorubicin, ROS reactive oxygen species, Tf transferrin, TfR transferrin receptor, GPX4 glutathione peroxidase 4, mPTP mitochondria permeability transition pore, Lipid-OO lipid peroxides, NLRP3 NOD−, LRR−, and pyrin domain-containing protein 3, TINCR terminal differentiation-induced ncRNA, GSDM-N gasdermin, TNFα tumor necrosis factor-alpha, TRADD tumor necrosis factor receptor type 1 associated death domain protein, FADD Fas-associated protein with death domain, RIPK receptor-interacting serine/threonine-protein kinase, CAMKII calcium/calmodulin-dependent protein kinase II, NRF2 nuclear factor erythroid 2-related factor, TFEB Transcription factor EB, NF-κΒ nuclear factor-κB, NFAT4 nuclear factor of activated T-cells, ROS reactive oxygen species, SIRT sirtuin, GATA4 GATA-binding protein 4.

Multiple RCD pathways are involved in doxorubicin’s effects on cardiomyocytes (Fig. 7). These multi-factorial mechanisms can occur simultaneously, are not independent, and may overlap or crosstalk, adding to the complexity of DIC. Sirtuins for example are involved in autophagy, apoptosis, and pyroptosis (Fig. 6), while also they have been associated with cell death due to iron overload as well^120^. Similarly, CAMKII activation leading to mPTP opening can occur in both necroptosis and apoptosis^59^. However, the involvement of identical molecules in multiple pathways may represent an opportunity as the same pharmacological agent can potentially block multiple pathways simultaneously. Berberine can activate SIRT1 which in turn can regulate autophagy, apoptosis, and pyroptosis^19,26,27,70^. While dexrazoxane was thought to only exert its cardioprotective effects through iron chelation, it has now been established that it works by blocking TOP2B as well^121^. As a result, understanding the crosstalk between RCD pathways will allow for the identification of therapeutic agents that target multiple pathways at once.

Understanding which RCD has the greatest effect in DIC would help prioritize which pathway to be preferentially blocked. Necrosis and necroptosis may have a greater responsibility in the pathogenesis of DIC compared to apoptosis^59^. However, ferroptosis and apoptosis also play important roles as well^54^. Evidently, identifying which pathways have the greatest responsibility in the pathogenesis of DIC requires further study.

Novel RCD pathways are continuously emerging and new key players within RCD pathways are also being discovered. Studying RCD pathways involved in DIC has led to significant advances in our understanding of the complex molecular pathogenesis of this important adverse drug reaction, and it is likely that there will be many future advances in this field. As our knowledge of the most important pathways involved in DIC continues to advance we will be better positioned to both test for cardiotoxicity and devise new approaches to protect against DIC.
